# A novel electron emission-based cell culture device promotes cell proliferation and differentiation of pre-osteoblastic MC3T3-E1 cells

**DOI:** 10.1371/journal.pone.0213579

**Published:** 2019-03-28

**Authors:** Fumiaki Sugimori, Hiroyuki Hirakawa, Ai Tsutsui, Hiroyuki Yamaji, Shohei Komaru, Mai Takasaki, Tadashi Iwamatsu, Toshimasa Uemura, Yo Uemura, Kenichi Morita, Takashi Tsumura

**Affiliations:** 1 Advanced Technology Development Unit, Business Solution Business Unit, SHARP CORPORATION, Yamatokoriyama, Nara, Japan; 2 Cell Culture Marketing & Research Center, JTEC COOPERATION, Ibaraki, Osaka, Japan; 3 Graduate School of Engineering, Osaka University, Suita, Osaka, Japan; University of Fukui, JAPAN

## Abstract

In this report we demonstrate the effect of a novel electron emission-based cell culture device on the proliferation and differentiation of pre-osteoblastic MC3T3-E1 cells. Our device has an electron emission element that allows, for the first time, stable emission of electrons into an atmosphere. Atmospheric electrons react with gas molecules to generate radicals and negative ions, which induce a variety of biochemical reactions in the attached cell culture system. In this study, we demonstrated the effect of this new electron emission-based cell culture device on cell proliferation and differentiation using pre-osteoblastic MC3T3-E1 cells. Electron emission stimulation (EES) was applied directly to culture medium containing plated cells, after which the number of living cells, the mRNA levels of osteogenesis-related genes, and the alkaline phosphatase (ALP) activity were evaluated. The growth rate of EES-exposed cells increased by approximately 20% in comparison with unexposed control cells. We also found the mRNA levels of osteogenic specific genes such as collagen type I α-1, core-binding factor α-1, and osteocalcin to be up-regulated following EES. ALP activity, a marker for osteogenic activity, was significantly enhanced in EES-treated cells. Furthermore, reactive oxygen species generated by EES were measured to determine their effect on MC3T3-E1 cells. These results suggest that our new electron emission-based cell culture device, while providing a relatively weak stimulus in comparison with atmospheric plasma systems, promotes cell proliferation and differentiation. This system is expected to find application in regenerative medicine, specifically in relation to bone regeneration.

## Introduction

Electron emission devices typically operate only in a vacuum and have long been used in vacuum tubes, CRTs, electron microscopes, and similar instruments. Comparatively few studies have been reported in which the operation of electron emission in an atmosphere was attempted. The MIS (Metal-Insulator-Semiconductor)-type electron emission device has been reported to operate from low vacuum up to atmospheric pressure [1,2]. However, its lifetime was too short to allow for stable operation due to rapid destruction or deterioration.

**Fig 1:**
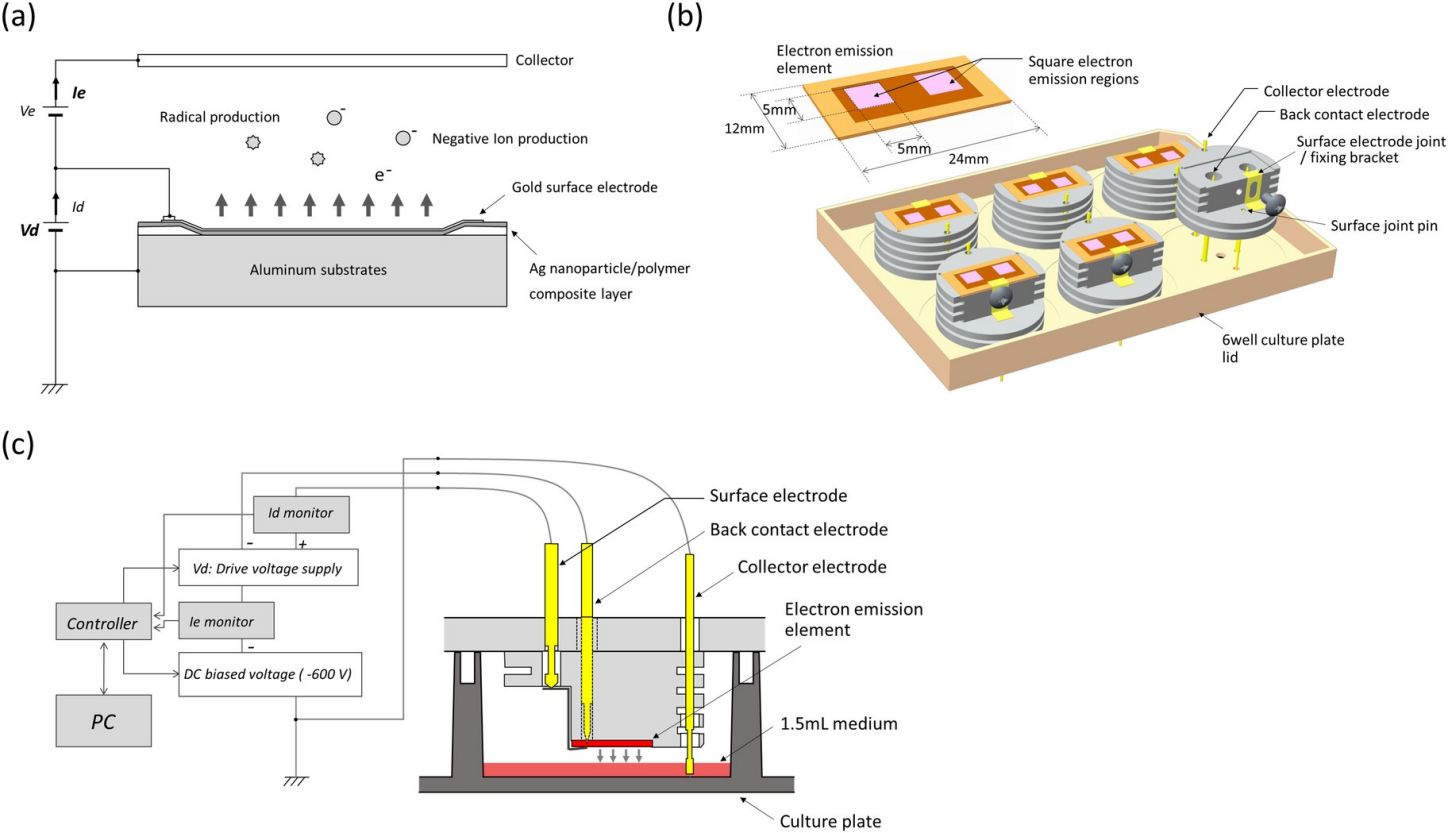
Our novel electron emission-based cell culture device and the EES system. (a) Schematic diagram of the electron emission element. The electron emission element emits electrons in the atmosphere when approximately 10–20 V (Vd: drive voltage) is applied. Emitted electrons fly to the collector along the electric field. The potential of the collector (Ve) is about 600 V higher than that of the element. The current in the element and the collector is expressed as Id and Ie, respectively. (b) 3D image of our novel electron emission-based cell culture device. The placement of six electron emission elements on an inverted modified 6-well culture plate lid is shown. An exploded view of the electron emission element highlights its components and their assembly, as well as the holes in the plate lid through which electrodes are inserted. (c) A principal schematic of the electron emission stimulation (EES) system. EES was given directly to cells in 1.5 ml of medium in the 6-well plate for 1 min. The stimulation condition can be modulated by a PC terminal through a controller.

In the process of developing a charger for an MFP (Multifunction Printer), our group at SHARP CORPORATION recently succeeded in developing the first electron emission device capable of stable operation in atmosphere [3]. Conventional chargers based on the discharge principle have the problem of generating toxic ozone and NOx, a problem solvable by electron emission in the atmosphere. Fig 1A shows a schematic illustrating the basic concept of our novel electron emission device. Briefly, an Ag nanoparticle/polymer composite layer was formed on an aluminum substrate with a thickness of about 1 μm, upon which a gold surface electrode with a thickness of 20 nm was then formed. Electrons can be emitted from the surface electrode by applying a voltage of approximately ten volts between the aluminum substrate and the surface electrode. The electrons released into the atmosphere generate negative ions and radicals, and the negative ions move to the collector electrode along the electric field. Our previous electron spin resonance (ESR) study using spin trap reagent 5,5-dimethyl-1-pyrroline-N-oxide (DMPO) showed that products generated from an electron emission device in the aqueous phase contained the hydroxyl radical (HO·), hydrogen radical (H·), and superoxide (O_2_^-^) [4]. Hydrogen peroxide (H_2_O_2_) was also detected, and it is considered that these reactive species are derived from O_2_ and H_2_O dissociated by ionized O_2_.

Many papers have been published regarding the effect of electrical stimulation on biological systems, including bone metabolism [5,6], skeletal muscle functions [7], the neural system [8], etc. Among these systems, electrical stimulation has found clinical application in the treatment of bone fractures. The indirect effects of electrical stimulation of the dorsal root ganglion [9,10] and to muscles [11] have been found to preserve bone mass. However, the direct effects of electrical stimulation on osteoblasts have also been observed in several studies [12–15]. While the major results from these manuscripts report positive effects of electrical stimulation on the proliferation and differentiation of cells, the details are complex and varied. This complexity might arise from the different ways in which electrical stimulation has been applied to the cells. Our novel electron emission-based cell culture device provides a relatively weak stimulus compared to existing atmospheric plasma systems [16]. This quality is expected to allow for the elucidation of the detailed mechanism underlying the electrical stimulation effect on cells. To illustrate its utility for this purpose, we chose a typical osteoblastic MC3T3-E1 cell line for the first application of our system to cultured cells. In this study, we introduce an electron emission system developed for application to cell culture and present findings from the application of this electron emission to MC3T3-E1 cells.

## Materials and methods

### Electron emission stimulation (EES) system

EES was conducted using our novel electron emission-based cell culture device as shown in Fig 1B. The outline and size of this device are almost the same as those of a 6-well culture plate lid. Each device has six electron emission elements, arranged on the inside of a 6-well culture plate lid, the surface of which is oriented toward the culture medium across an air gap. The dimensions of the electron emission element are 24 mm × 12 mm, containing two 5-mm square electron emission regions. Electrons are emitted from these square regions. Gold electrodes were fixed near the elements. On the back of the electron emission element, DC bias voltage (-600 V) was applied through a back contact electrode as shown in Fig 1C. A 13.5 ~ 22.0 V higher voltage was applied on the front than on the back surface, that is, -578 ~ -586.5 V. This potential difference (13.5 ~ 22.0 V) is defined as a drive voltage (Vd [V]), whose waveform and frequency are rectangular pulses and 528 Hz, respectively. The current inside the element (Id) was monitored and kept under a predetermined threshold so as not to overheat the medium. The gold collector electrode was grounded and soaked in the culture medium, causing the culture medium to also be grounded. The electron emission elements emitted electrons to the medium depending on the electric field generated between the elements and medium (the air gap measures approximately 1 mm). These electrons are collected through the collector electrode, with the amount expressed as electron emission current (Ie [μA]). The value of Ie was monitored and kept constant by controlling duty cycle. In this study, we modulated Vd and Ie as key parameters and investigated their effect on cellular proliferation and differentiation of MC3T3-E1 cells.

### Cell culture

MC3T3-E1 cells were obtained from the American Type Culture Collection (ATCC, Rockville, MD, USA) and maintained in alpha-modified Eagle’s medium (α-MEM; Gibco BRL, Rockville, MD, USA) with 10% heat-inactivated fetal bovine serum (FBS; Sigma-Aldrich, St. Louis, MO, USA) and 1% antibiotic-antimycotic (Gibco).

Cells at passages 4–6 were grown in 6-well cell culture plates (AS ONE, Osaka, Japan) at initial seeding cell densities of 50,000 cells/well under controlled conditions of temperature at 37°C and a humidified atmosphere of 95% air and 5% CO_2_. Cells were subjected to EES for 1 min per day in an incubator 1 and 2 days after seeding (day 0 and day 1, respectively) and harvested at day 2, 3, 6, and 9. Medium was replaced with fresh medium every 3 days.

### Cell proliferation assay

Cells were subjected to EES for 1 min per day on day 0 and day 1, harvested on day 2, and the living cells enumerated. First, cells were washed with 1 ml phosphate buffered saline (PBS; Wako Pure Chemical Industries, Ltd., Osaka, Japan) per well twice, after which 400 μl of trypsin-EDTA solution (Sigma-Aldrich) was added to each well and plates were incubated at 37°C for 5 min. After incubation, 1 ml of growth medium (described above) was added to each well, and the number of living cells was determined using a LUNA^TM^ Automated Cell Counter (Logos Biosystems, Inc., Annandale, VA, USA).

### Detection of reactive oxygen species (ROS) by EES

The concentration of H_2_O_2_ in pure water and culture medium generated by EES treatment was measured using the Amplex Red probe (Invitrogen, Carlsbad, CA, USA). EES treatment was performed on 1.5 ml pure water or culture medium without cells, and 50 μl of the treated solutions was transferred to individual wells of a 96-well transparent plate. To generate a standard curve, H_2_O_2_ solution of 0 to 10 μM was prepared. After adding the Amplex Red reagent to each well, the microplate was incubated at room temperature for 30 min. Then, fluorescence was measured using a multiwell plate reader (TECAN Infinite 200 PRO, Männedorf, Switzerland) with excitation/emission filters of 550/585 nm.

Detection of other intracellularly induced ROS was also performed using fluorescent probes. Cells were cultured for 3 days and then stained with OxiOrange (excitation: 543 nm, emission: 577 nm; Goryo Chemical, Sapporo, Japan) or HySOx (excitation: 555 nm, emission: 575 nm; Goryo Chemical), which react with intracellular HO· and hypochlorous acid (HClO), respectively, and emit strong fluorescence. After staining for 30 min at 37°C, cells were washed with PBS, and EES treatment was conducted with 0.5 ml culture medium, and then intracellularly induced ROS were observed by fluorescence microscopy (Axio Observer, Carl Zeiss, Jena, Germany). Nuclei were stained with DAPI (4′6-diamidino-2-phenylindole).

### RNA extraction and quantitative RT-PCR analysis

Total RNA was purified using an RNeasy Mini kit (QIAGEN GmbH, Hilden, Germany). The RNA concentration and purity were measured by Nanodrop Assay (Thermo Fisher Scientific, Waltham, MA, USA). For cDNA synthesis, RNA was reverse transcribed using a PrimeScript^TM^ RT reagent Kit (Takara Bio Inc., Shiga, Japan). For quantitative RT-PCR analysis, gene-specific primers including Gapdh (glyceraldehyde-3-phosphate dehydrogenase), Col1a1 (collagen Type I α-1), Spp1 (osteopontin), Ocn (osteocalcin), and Cbfa1 (core-binding factor α-1), and TGF-β1 (transforming growth factor beta 1) were designed for use with TaqMan Gene Expression Assays (Applied Biosystems Japan Ltd., Tokyo, Japan; Gapdh: Mm99999915_g1, Col I: Mm00801666_g1, Spp1: Mm00436767_m1, Ocn: Mm03413826_mH, Cbfa1: Mm00501584_m1, and TGF-β1: Mm00498234_m1). The qPCR amplification was carried out as follows: initial heating at 95°C for 20 s, followed by 40 cycles of denaturation at 95°C for 1 s, and annealing at 60°C for 20 s. Gene copy numbers were measured in triplicate and normalized against the average copy number for Gapdh.

### Alkaline phosphatase (ALP) activity assays and staining

After the EES treatment described above, the cells were assayed for ALP activity by means of a spectrophotometric method using a LabAssay^TM^ ALP kit (Wako). Cells were lysed in a lysis buffer (Wako), incubated for 10 min on ice, and then centrifuged at 20,000 *g* for 30 min at 4°C. The clear cell lysate was transferred to a new 1.5 ml centrifuge tube for the ALP assay. Cell lysate (20 μl) was added into each well of a 96-well transparent plate. Following this, 100 μl of ALP substrate solution was added to each well, and the plate was incubated in the dark for approximately 15 min at 37°C. After incubation, the absorbance was read at 405 nm using a multiwell plate reader (TECAN Infinite 200 PRO). Each value was normalized against the DNA content, determined using a PicoGreen dsDNA Kit (Invitrogen). The enzymatic activity was expressed as pmoles of p-NP/min/μg DNA.

ALP staining of the cells was performed as follows. The cells were washed by PBS twice and fixed by 3.7% paraformaldehyde (PFA). Then, cells were stained with the TRAP/ALP Stain Kit (Wako) according to the manufacturer’s protocol. Stained cells were observed using the CC-5000 Cell3 iMager (SCREEN, Kyoto, Japan).

### Statistical analysis

Average values for ALP activity, mRNA expression of Col 1, Spp1, Ocn, Cbfa1, and TGF-β1, H_2_O_2_ concentration, and the number of living cells were calculated, expressed as the arithmetic mean ± S.D., and plotted on graphs. A paired *t* test was used for statistical evaluation. The statistically significant differences between the experimental and control groups were established at the p < 0.05 (*) and p < 0.01 (**) levels.

## Results

### Production of ROS by EES

**Fig 2:**
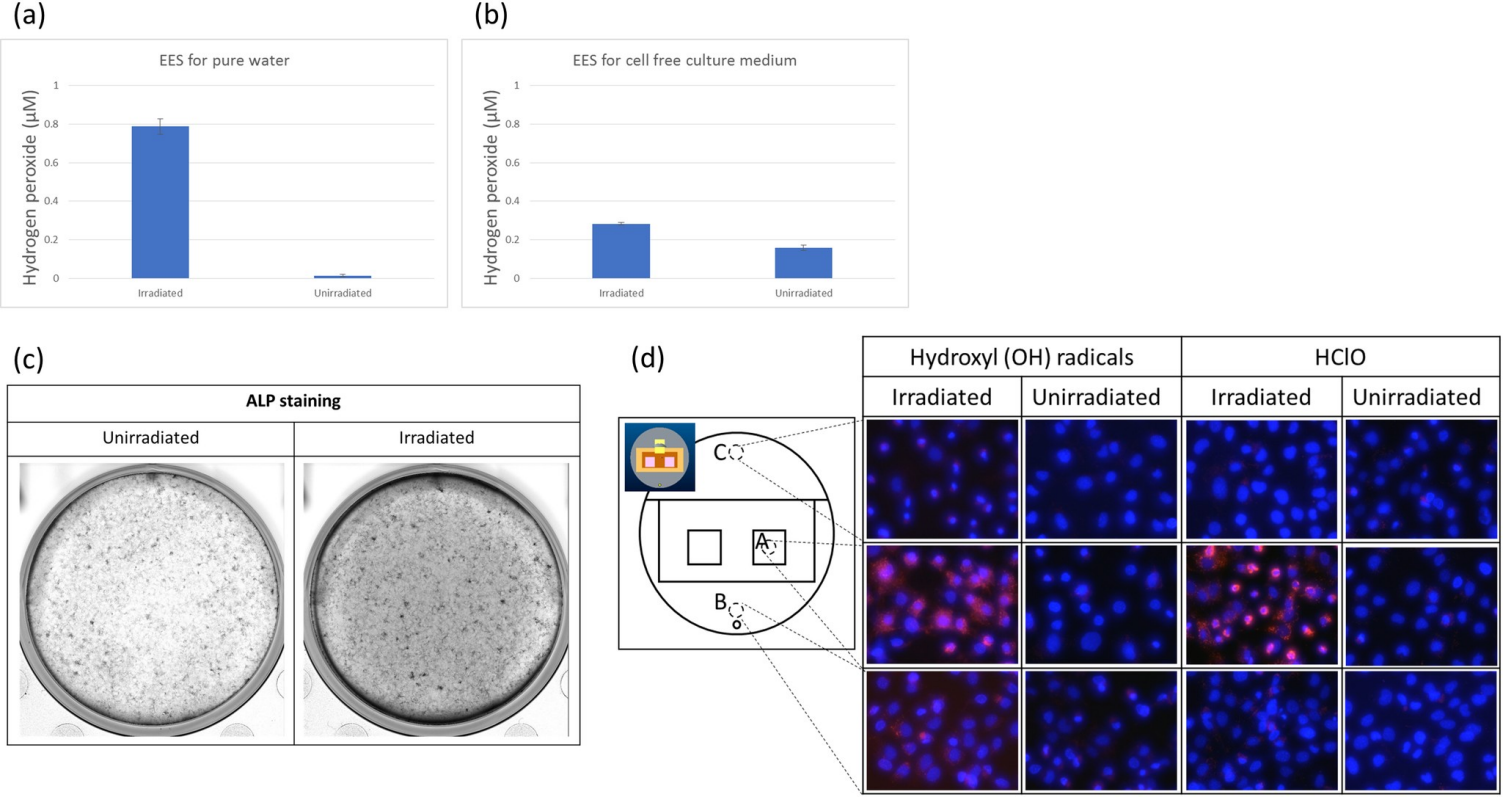
ROS production by EES. (a) H_2_O_2_ concentration in pure water with or without EES treatment. Vd and Ie were fixed at 15.0 V and 2.3 μA, respectively, and irradiation time was 1 min. The data points represent the mean value ± SD (n = 3). (b) An experiment similar to that in (a) was conducted for culture medium without cells. (c) Alkaline phosphatase staining of the MC3T3-E1 cells with no stimulation and subjected to EES (Vd and Ie were fixed at 15.0 V and 2.3 μA respectively), for 4 days. (d) Images of fluorescent compounds produced by reaction with intracellularly induced HO· and HClO (both are red) by EES. A: immediately under the irradiation position, B: just beside the collector electrode, C: far away from the irradiation position. Nuclei were stained with DAPI (blue).

First, we performed EES treatment of pure water and medium and measured the concentration of H_2_O_2_ contained therein. For this measurement, element Vd was set to 15.0 V, Ie was 2.3 μA, and irradiation time was 1 min. After EES treatment, the concentration of H_2_O_2_ was 0.8 μM in pure water and 0.1 μM in culture medium (Fig 2A and 2B). Although this was a much lower concentration than that obtained by plasma irradiation, the number of dead cells increased when irradiated for more than 1 min; therefore, the stimulation time was set to 1 min in subsequent experiments.

The results of ALP staining are shown in Fig 2C. The EES-treated cells showed stronger staining than untreated cells. However, the staining was not specifically stronger under the electron emission element but almost homogeneous as shown in Fig 2C. Next, we observed intracellularly induced HO· and HClO immediately after EES treatment. In this experiment, Vd was set to 15.0 V, and Ie was 5.0 μA. As shown in Fig 2D, cells in the neighboring region under the electron emission element more strongly expressed HO· and HClO compared with other areas when the medium depth was shortened (0.5 ml medium/well) compared with the normal experimental condition (1.5 ml medium/well). These results suggested that the ROS including HO· or HClO generated by EES might be key factors that promote the differentiation of MC3T3-E1 cells. They may diffuse in the medium by Brownian motion of the H_2_O molecules. The larger the depth of medium, the less uniform the differentiation of osteoblasts by EES.

### Cell proliferation

**Fig 3:**
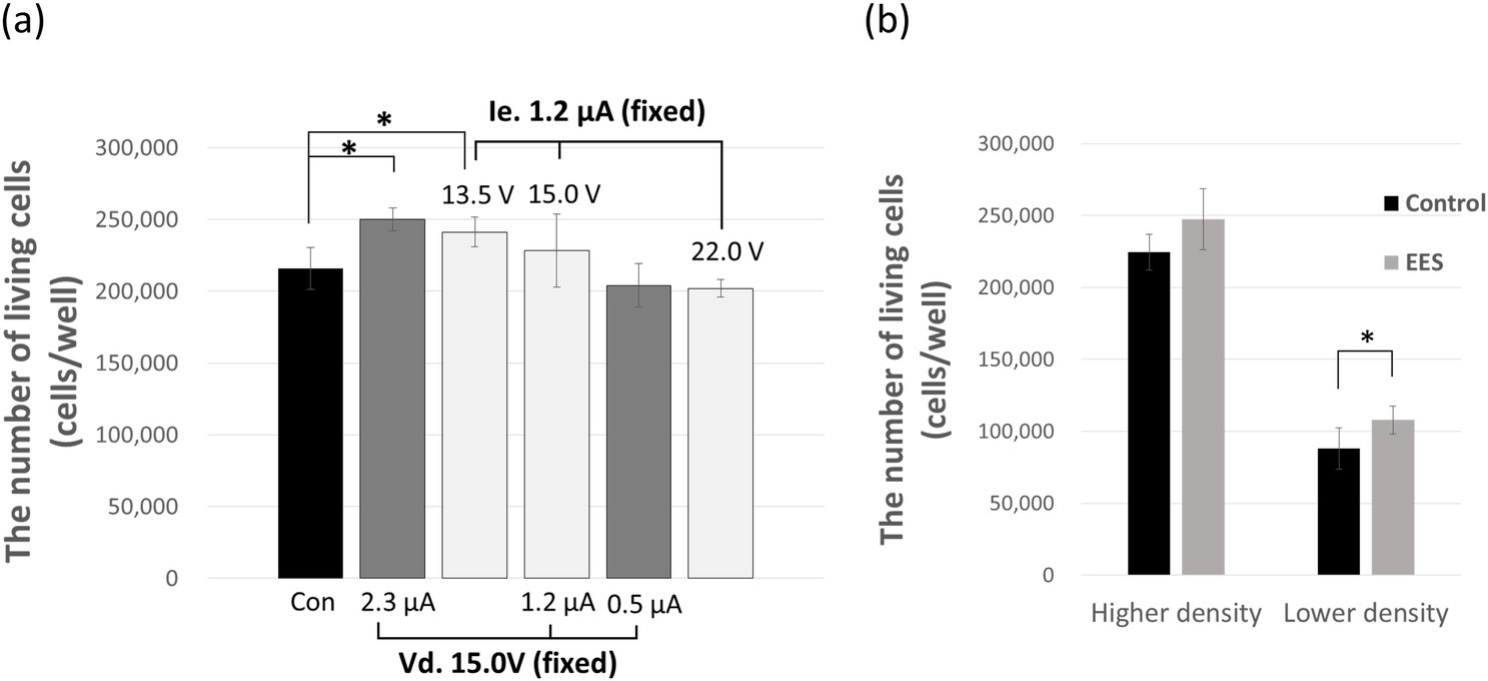
Effect of EES on the proliferation of MC3T3-E1 cells. (a) MC3T3-E1 cells were subjected to EES for 2 days, modulating Vd or Ie, and the number of living cells were counted at day 2. The data points represent the mean value ± SD (n = 3), *p < 0.05 vs. control. (b) MC3T3-E1 cells with higher and lower seeding density were subjected to EES. Conditions of electron emission were the same as in Fig 3A. The data points represent the mean value ± SD (n = 3), *p < 0.05 vs. control.

The effect of EES on MC3T3-E1 cell proliferation was analyzed by comparing the number of living cells treated by EES with the control group (cultured normally without EES). We conducted an EES treatment in which we modulated (Vd) and (Ie) to assess how these parameters could affect cell proliferation (Fig 3A). After 2 days of EES treatment, Cell numbers were determined using an automated cell counter. When the drive voltage was fixed at 15.0 V and the electron emission current was modulated from 0.5 μA to 2.3 μA, the cell proliferation rate increased with increases in current. On the other hand, when electron emission current was fixed at 1.2 μA and the drive voltage was modulated from 13.5 V to 22.0 V, the cell proliferation decreased as the drive voltage increased. Cell proliferation maximized at approximately 116% of that in the control group when the drive voltage was 13.5 V and electron emission current was 1.2 μA (p < 0.05). We also studied the effect of cell seeding density on the proliferation rate. Compared to higher density (seeding density; 50,000 cells), lower density seeding (25,000 cells) showed more a significant difference of proliferation rate by EES, in which the EES group showed 22% more than the control group (Fig 3B). This result indicated that the combination of lower drive voltage and higher electron emission current in the measured range promoted cell proliferation.

Comparing the electron emission current and the drive voltage of the system, the emission current per cell might be more important than the drive voltage considering the effect of seeding density on the proliferation rate, suggesting a possible mechanism underlying the effect of EES on cells.

### Gene expression analysis

**Fig 4:**
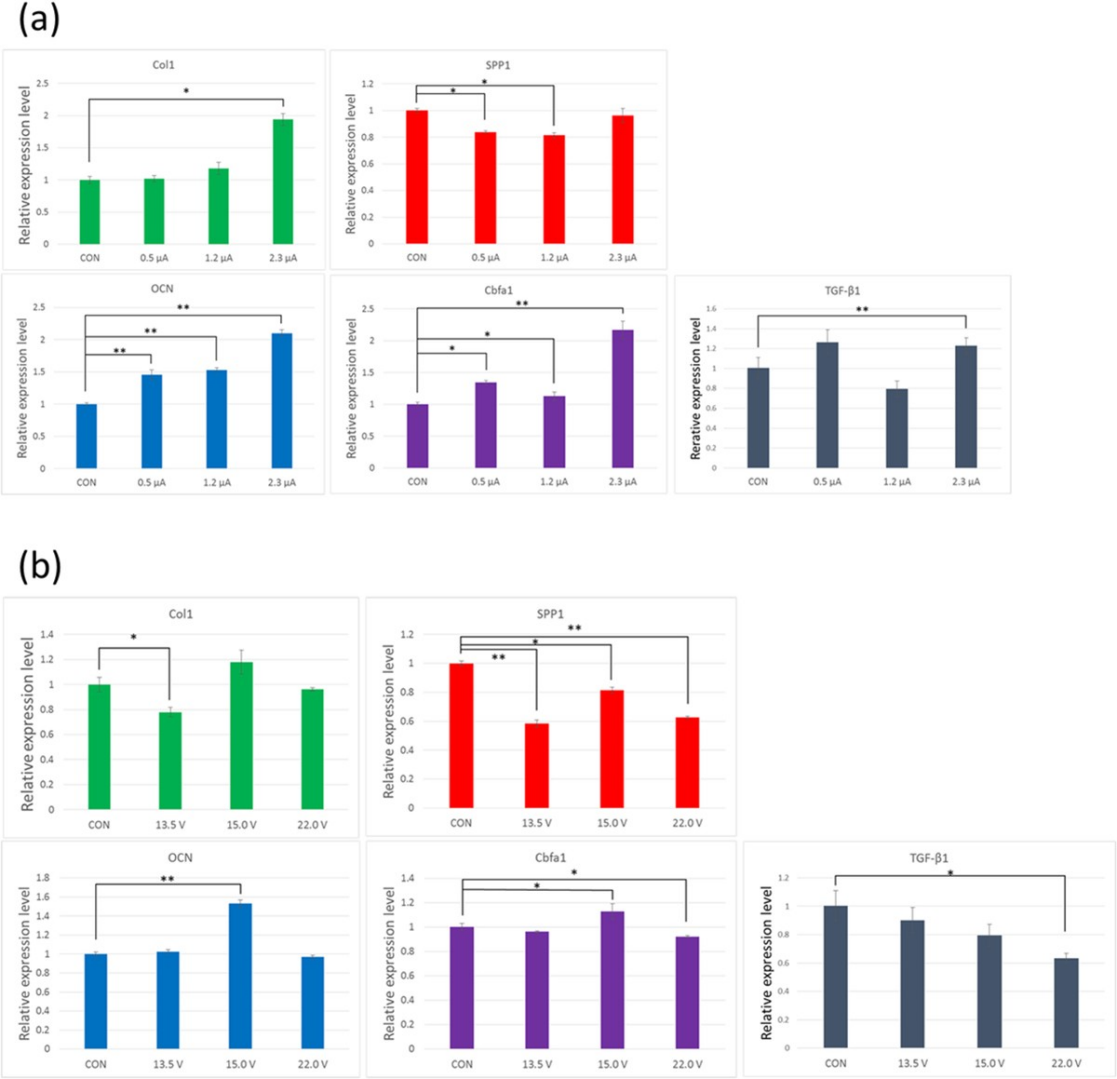
Gene expression of osteogenesis-related genes. (a) MC-3T3-E1 cells were subjected to EES for 2 days (Vd was fixed at 15.0 V and Ie was modulated). The mRNA levels of collagen I (Col1), osteopontin (Spp1), Ocn (osteocalcin), Cbfa1, and transforming growth factor beta 1 (TGF-β1) were quantified by real-time PCR. The data points represent the mean value ± SD (n = 3), *p < 0.05, **p < 0.01 vs. control (CON). (b) The experimental conditions were almost the same as those in Fig 3A except that Ie was fixed at 1.2 μA and Vd was modulated.

To study whether our EES device had the potential to induce cell differentiation, we evaluated the mRNA expression levels of the osteogenesis-related Col1a1, Cbfa1, Spp1, Ocn, and TGF-β1 genes in pre-osteoblast MC3T3-E1 cells. The experimental conditions were the same as those used in the cell proliferation assay. When the drive voltage was fixed, and the electron emission current modulated, the expression of Col I, Cbfa1, and Ocn increased as the current was increased (Fig 4A). The expression of TGF-β1 increased in a similar manner to Cbfa1. When the current was fixed and the drive voltage was modulated, Col I, Cbfa1, and Ocn expression was maximized at 15.0 V, while at 13.5 V or 22.0 V they were not obviously different from the expression levels observed in the control group (Fig 4B). With the drive voltage at 15.0 V and electron emission current at 2.3 μA (Fig 4A), the mRNA levels of those genes were significantly higher, approximately twice the levels found in the control group. The expression of TGF-β1 decreased with the drive voltage (Fig 4B). Spp1 differed from the other genes in terms of its electron emission current and drive voltage dependence, with the former having little influence on its expression (Fig 4A), while, in the latter case, the influence was more complex (Fig 4B). Gene expression analysis of Col I, Cbfa1, Ocn, and TGF-β1 in MC3T3-E1 cells suggested that our new device was able to induce osteoblastic differentiation under conditions in which the drive voltage was set to 15.0 V, and at high electron emission current.

### ALP activity

**Fig 5:**
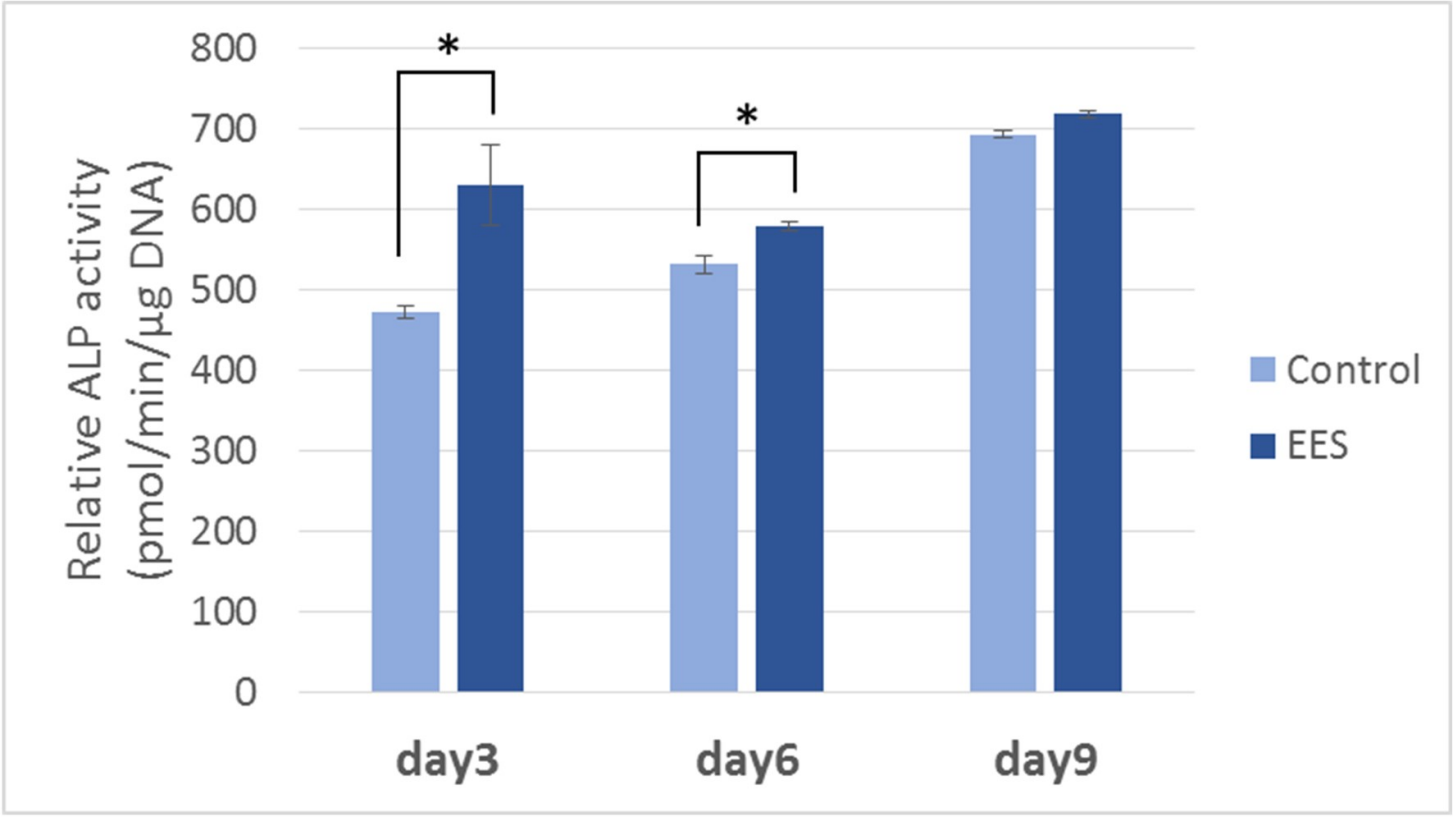
Effects of EES on the ALP activity of MC3T3-E1. Cells were subjected to EES for 2 days (Vd and Ie were fixed at 15.0 V and 1.2 μA, respectively), and the ALP activities were examined at day 3, 6, and 9. The data points represent the mean value ± SD (n = 3), *p < 0.05 vs. control.

ALP activity in MC3T3-E1 cells was examined to evaluate the effect of EES on osteogenic cellular differentiation. For this measurement, drive voltage was set to 15.0 V, and the electron emission current was set to 1.2 μA. After 2 days of EES treatment, cells were harvested on days 3, 6, and 9, and the ALP activity in MC3T3-E1 cells was measured in relation to their DNA content. On day 3, the ALP activity in EES treated cells was more than 30% (p < 0.05) higher than in non-treated control cells. On day 6, the ALP activity was 8.7% higher (p < 0.05) than in the control (Fig 5). However, on day 9, the ALP activity did not differ from that in the control cells. These results demonstrated that EES induced osteogenic differentiation of MC3T3-E1 cells, but that the effect of EES treatment on ALP activity, while detectable for approximately a week, was not maintained over the long term.

## Discussion

Our study demonstrated the effect of an electron emission-based cell culture device on the proliferation and differentiation of pre-osteoblastic MC3T3-E1 cells.

Previous studies have investigated the effect of electrical stimulation on bone metabolism [5,6]. Clinically, electrical stimulation has been applied for the treatment of bone fractures. Electrical stimulation to the dorsal root ganglion [9,10] or to muscle [11] has the indirect effect of preserving bone mass. However, the effects of direct electrical stimulation on osteoblasts have also been described in several studies [12–15]. Furthermore, the osteoblastic differentiation of mesenchymal stem cells under an electrical field has been studied [16]. The major results from these studies are supportive of the conclusion that electrical stimulation has a positive effect on cellular proliferation and differentiation. However, the details are complex, with results sometimes being dependent on the stage of differentiation. Furthermore, in some cases the effect on proliferation appears to be greater than the effect on differentiation, while in others the reverse is true, as seen in one comparative study of SaOS-2 and MC3T3-E1 [14]. Such complexity might arise from the different ways in which electrical stimulation has been applied to the cells. Different systems form different spatial electric fields and current flow in the cell culture system, meaning that the stimulation applied to the cells depends on the system and includes variables such as applied voltage, current, frequency, application period, etc. The biological effects of electron emission may be categorized in terms of reactive product induction and signal transduction via channels. Previously, we measured the concentrations of some reactive products including ROS induced by our system in cell-free pure water in a similar experiment. O_2_^-^ ions were detected in the region between the electrode and liquid surface (data not shown). An electron spin resonance study using the spin trap method showed the presence of HO·, H·, and O_2_-, whose concentration was approximately 2.0, 1.0, and 0.3 μM, respectively, in the liquid after 60 min electric emission with an applied voltage of about 20 V [4]. The present study was performed with 1 min electron emission, and the estimated concentrations were much lower than these values. The concentration of H_2_O_2_ was 0.8 μM in pure water and 0.1 μM in culture medium with 1 min electron emission of 2.5 μA electric current. Thus, the concentration of radicals (for example ROS) is estimated to be similar to 0.1 μM, based on the theory of radical formation by X-ray irradiation of water. The concentration of radicals induced by our system is approximately 100-fold smaller than that induced by atmospheric plasma systems. For example, the concentration of H_2_O_2_ formed by He atmospheric-pressure plasma (He-APP) irradiation is in the order of 10 μM [17,18]. The concentration of ROS in our system was two orders smaller than that in the He-APP system; however, detection of HO· and H_2_O_2_ in MC3T3-E1 cells clearly indicated the cytosolic capture of HO· and H_2_O_2_ emitted from the electrode via culture medium as shown in Fig 2D. ROS are known to play a role in attenuating proliferation, arresting the cell cycle, and increasing sensitivity to apoptosis. Nonetheless, this study demonstrated the positive function of ROS produced by electron emission in not only proliferation but also osteogenic differentiation of MC3T3-E1 cells. Similarly, He-APP [18] has been reported to induce osteoblastic differentiation of MC3T3-E1 cells, but these results do not explain the irradiation time dependence on ALP activity due to the toxicity of ROS at high concentration. Our system produces a much lower concentration of ROS than the He-APP system. Furthermore, the electric current dependence on mRNA of Col I, Ocn, and Cbfa1 is reasonably explained in our system. We measured the effects of electron emission energy and electron emission current on the proliferation of MC3T3-E1. The proliferation rate was not affected by increasing energy, but did increase when the current was increased (Fig 3A). Regarding generation of reactive products, by increasing electron emission current with fixed emission energy (voltage), the number of reactive products with similar composition is expected to increase, which might affect the proliferation. On the contrary, by increasing emission energy with fixed emission current, the same number of reactive products are voltage-dependently increased but with altered composition. According to our preliminary study, the increase in emission energy (voltage) induced a rapid increase in NO_2_^-^ and increase in NO_3_^-^, molecules which might attenuate the proliferation. Furthermore, Fig 3B shows the marked differences in proliferation rate arising from electrical stimulation of cells with low and high cell seeding densities. This indicated that the number of generated reactive products per cell had an important effect on proliferation, which supports the idea of signal promotion in the cells. This explanation is also applicable to the qPCR results in Fig 4, which shows the mRNA expressions of five kinds of osteoblastic markers, collagen type I, osteopontin, osteocalcin, cbfa1, and TGF-β1, under two variations of applied voltage and current, variable voltage and fixed current density, and variable current density and fixed voltage. When the current density increased on the fixed voltage, the expression of collagen type I, osteocalcin, cbfa1, and TGF-β1 increased almost monotonously; however, that of osteopontin was not highly dependent on the current density. On the contrary, the voltage dependence of each marker gene is complex, as shown in Fig 4B. The expression of TGF-β1 decreased in a voltage-dependent manner, which might be caused by the same mechanism of voltage-dependent proliferation due to a rapid increase in NO_2_^-^ (Fig 2A). Other markers displayed complex behavior. These results also support the importance of current density, which indicates the number of emitted reactive products per cell.

Fig 5 shows that electron emission stimulated ALP activity in MC3T3-E1 cells. At day 3, ALP activity following EES treatment was 30% higher than in the untreated control. This result confirmed that EES treatment promoted the osteoblastic differentiation of MC3T3-E1 cells. However, differences in ALP gradually decreased as culture time increased, and by day 9 ALP was only 5% higher in treated cells than in the control. In our study, osteoblastic markers, namely the mRNA expression levels of the collagen type I, osteocalcin, and core-binding factor α-1 genes, were up-regulated by an increase of the current density on fixed voltage, while the expression of osteopontin showed little response to changes in current density. Collagen type I is related to both cell proliferation and differentiation, because it plays a role not only in cell adhesion, but also in hydroxyapatite formation. Osteocalcin plays an important role in the late stage of osteogenesis by osteoblasts, which secrete osteocalcin-rich bone matrices. Cbfa1 (core binding factor α-1) is an essential transcription factor for the osteogenic lineages and is indispensable for *in vivo* bone formation [19–21]. These factors play a direct role in the osteogenesis of osteoblasts, while osteopontin plays an indirect role in the bone formation and bone remodeling process. Osteopontin is a type of non-collagenous matrix protein, which is secreted by osteoblasts as well as osteoclasts. It enables the binding of osteoblasts to hydroxyapatites and is also responsible for osteoclasts anchoring to bone matrices in the bone remodeling process [22,23]. Thus, the effect of EES might relate only to the direct process of osteogenesis by osteoblasts.

The fate of electrons emitted from the electrode of EES system in the culture dish is unclear. They might affect some signal transduction pathway. As described above, between the electrode and medium surface, O_2_^-^ molecules were prevalent. Under the medium surface, reactive products including HO·, H·, O_2_^-^, and H_2_O_2_ were detected by spin trap experiments. Furthermore, the cytosolic capture of HO· and H_2_O_2_ emitted from the electrode via culture medium in MC3T3-E1 cells was detected. These results suggest that some reactive products promoted the osteoblastic differentiation of MC3T3-E1 cells, but the predominant contributor remains unclear. Based on the results in Fig 3A, the emission current dependence of Cbfa1 and TGF-β1 was similar. TGF-β1 has been reported to promote osteoblastic differentiation of MC3T3-E1 cells via the calcium/calmodulin pathway following electric stimulation and to target Runx2 (Cbfa1) in cooperation with BMP2 for osteoblastic differentiation of pluripotent mesenchymal precursor cell line C2C12 [24]. Calcium channels may potentially play a role in the effect of EES on the fate of cells. The reactive products might trigger the physiologically relevant Ca^2+^ influx through Ca^2+^ channels. Among them, the TRP (transient receptor potential) channel family is a possible candidate [17].

Some reports have described the effects of EES systems on MC3T3-E1 cells [17,18,25–26]. All of the studies suggested a positive effect of EES on osteoblastic differentiation of MC3T3-E1 cells. Among them, two show an electric stimulation system in which the electrodes are in direct contact with the culture medium [25,27]. Other studies [17,18,26] demonstrated the use of an electric stimulation system with plasma radiation. The former [25,27] suggests that the ions generated from electrodes might affect the cells; on the contrary, the latter system [17,18,26] might regenerate reactive products that interact with cells. Our system is categorized as the latter, and the concentration of reactive products is expected to be one order smaller than that in the system involving plasma irradiation. Thus, our system is considered to be much safer than the plasma systems.

In conclusion, we report a novel electron emission-based cell culture device that promotes proliferation and induces differentiation of pre-osteoblastic MC3T3-E1 cells, through variation of drive voltage and electron emission current. An increase of electron emission current with fixed drive voltage promoted both cellular proliferation and differentiation, but as electron energy increased, the proliferation rate decreased, and the expression level of osteogenic marker genes have a maximum peak in the measured range (Table 1). Another point is that, based on our analysis of ALP activity, the effect of EES on cells was maintained for approximately a week, after which it declined to basal levels. We propose that this novel cell culture apparatus might have clinical potential for accelerating regenerative medicine, such as bone regeneration. Further study should be undertaken to clarify the biochemical and electrochemical mechanisms underlying the effects of EES on MC3T3-E1 cells.

**Table 1:** Summary of the effect of EES on the proliferation and differentiation of MC3T3-E1 cells.

EES Conditions	Proliferation rate	Differentiation rate
Ie: Increase (Vd: fixed)	Increase	Increase
Vd: Increase (Ie: fixed)	Decrease	Increase then Decrease

## Abbreviations

EES: electron emission stimulation
EDTA: ethylenediaminetetraacetic acid
ALP: alkaline phosphatase
MFP: multifunction printer
